# Photoacoustic Mouse Brain Imaging Using an Optical Fabry-Pérot Interferometric Ultrasound Sensor

**DOI:** 10.3389/fnins.2021.672788

**Published:** 2021-05-17

**Authors:** Yuwen Chen, Buhua Chen, Tengfei Yu, Lu Yin, Mingjian Sun, Wen He, Cheng Ma

**Affiliations:** ^1^Department of Electronic Engineering, Tsinghua University, Beijing, China; ^2^Department of Ultrasound, Beijing Tiantan Hospital, Capital Medical University, Beijing, China; ^3^School of Information Science and Engineering, Harbin Institute of Technology, Weihai, China; ^4^School of Astronautics, Harbin Institute of Technology, Harbin, China; ^5^Beijing National Research Center for Information Science and Technology, Beijing, China; ^6^Beijing Innovation Center for Future Chip, Beijing, China

**Keywords:** photoacoustic, mesoscopy, brain imaging, Fabry-Pérot interferometer, multiwavelength imaging

## Abstract

Photoacoustic (PA, or optoacoustic, OA) mesoscopy is a powerful tool for mouse cerebral imaging, which offers high resolution three-dimensional (3D) images with optical absorption contrast inside the optically turbid brain. The image quality of a PA mesoscope relies on the ultrasonic transducer which detects the PA signals. An all-optical ultrasound sensor based on a Fabry-Pérot (FP) polymer cavity has the following advantages: broadband frequency response, wide angular coverage and small footprint. Here, we present 3D PA mesoscope for mouse brain imaging using such an optical sensor. A heating laser was used to stabilize the sensor’s cavity length during the imaging process. To acquire data for a 3D angiogram of the mouse brain, the sensor was mounted on a translation stage and raster scanned. 3D images of the mouse brain vasculature were reconstructed which showed cerebrovascular structure up to a depth of 8 mm with high quality. Imaging segmentation and dual wavelength imaging were performed to demonstrate the potential of the system in preclinical brain research.

## Introduction

The rodent brain is an important model for the study of human brain diseases. Many neurologic diseases including stroke, Alzheimer’s, and brain tumors can alter the structure and function of the brain (Hirsch et al., 2012; Xie et al., 2012). The structural and functional changes of the brain vasculature can be key indicators of disease progression and treatment. The whole mouse brain vasculature can been imaged using biomedical imaging modalities such as magnetic resonance imaging (MRI) and micro computed tomography (micro CT) *in vivo* (Beckmann et al., 1999; Reese et al., 1999; Beckmann, 2000; Ritman, 2011; Clark and Badea, 2014). For higher resolutions, optical microscopy, such as micro-optical sectioning tomography (MOST), can be used for post-mortem imaging (Xiong et al., 2017; Zhang et al., 2019).

MRI angiographic imaging uses contrast agents to visualize small vessels *in vivo*, but the injected contrast agent may affect brain function (Bolan et al., 2006). Micro CT also faces the same problem but it can reach higher resolution. Resolution as high as 19 μm was reported *in vivo* with a long circulating liposomal-iodinated contrast agent (Starosolski et al., 2015). Both micro CT and MRI equipment are costly and bulky. Ultrasound imaging is emerging as a powerful tool for structural and functional imaging of the rodent brain, yet microbubbles injection is needed to obtain high resolution, and ultrasound imaging intrinsically lacks molecular contrast (Errico et al., 2015; Sieu et al., 2015; Urban et al., 2015; Demené et al., 2016). Optical imaging methods including near-infrared II fluorescence imaging (NIR-II) and two photon microscopy were also used to observe cerebrovascular structure with high resolution (Sakadžiæ et al., 2010; Hong et al., 2012). But most of these methods focus on the cortex because of limited penetration depth. The maximum depth of about 3 mm achieved by NIR-II microscopy is still far from covering the whole brain (Hong et al., 2014). Photoacoustic (PA) imaging is a hybrid modality that converts absorbed optical energy into ultrasound emission, thus a PA image shows optical absorption contrast and ultrasonic resolution. PA imaging inherits the advantages of optical and ultrasound imaging—its absorption contrast leads to high sensitivity to blood, making label-free angiography possible; using ultrasound for signal localization allows for deep imaging depth. Moreover, since the absorption spectrum of hemoglobin changes with oxygen saturation (sO_2_), PA can measure the sO_2_ distribution via spectroscopic imaging (Zhang et al., 2006; Tzoumas et al., 2016).

In PA imaging, the ultrasound transducer is a key element to ensure the quality of the signal. Despite the capability of parallel detection to enable fast data acquisition, traditional piezoelectric transducers (PZT) have some drawbacks when applied to PA imaging (Kong and Hwang, 1990; Lin et al., 2021). First of all, the bandwidth of PZT is limited, resulting in a loss of image features. Secondly, the poor angular coverage generates a missing cone complementary to the covered solid angle. Unlike in traditional optical imaging, the missing cone not only blurs the image, but also makes certain features completely invisible because PA signals are feature-dependent and highly directional. This problem is termed the “limited view problem” (Tao and Liu, 2010). The problem can be mitigated by rotating a single-element transducer or a linear array, or by using a ring-shaped array. Nevertheless, to ensure a reasonable field of view (FOV), the PZT-based sensors need to be placed at a great distance from the mouse head, creating complications such as high water pressure. PA microscopy (PAM) systems, including optical resolution (OR-) and acoustic resolution (AR-) PAMs, are more convenient to use when the mouse is in its natural prone position. Both of them can offer high resolution and sO_2_ information (lateral ~5 μm, axial 15 μm for OR-PAM and 50~100 μm for AR-PAM), but their penetration depth is limited (no more than 3 mm) and only vessels on the cortex can be observed (Yao and Wang, 2014; Yao et al., 2015). Recently, ultra-wide band ultrasound transducers have been employed for PA mesoscopy. These sensors provide a bandwidth beyond 100 MHz, and relatively broad angular coverage, thus could produce 3D photoacoustic images with fine details. Despite the superior image quality, the transducer’s relatively large weight and size may potentially pose challenges for fast scanning, paralleled detection, and optical illumination (Omar et al., 2013, 2014, 2019; Aguirre et al., 2017).

Ultrasound sensors based on optical detection can alleviate these problems. In particular, high finesse Fabry-Pérot interferometric (FPI) sensors have been implemented in photoacoustic computed tomography (PACT) to provide unprecedented signal quality (Jathoul et al., 2015). The planar FPI fabricated by Zhang et al. (2008) had a noise equivalent pressure (NEP) of 210 Pa over 20 MHz bandwidth and the element size was defined by the optical spot size to be 8 μm, resulting in a wide angular coverage. The sensor was demonstrated useful in mouse brain imaging (Laufer et al., 2009). The sensor’s sensitivity was limited by the relatively low finesse due to the “walk off” effect inside the cavity. To enhance the cavity finesse, plano-concave cavities, either fabricated on a planar glass or a fiber tip, were proposed (Guggenheim et al., 2017). The high-quality factor renders the sensor’s resonant wavelength susceptible to environmental conditions such as temperature change. As a result, the interrogation laser must have very narrow bandwidth, very low noise, and wavelength tuning capability. The high cost of such lasers prevents paralleled detection if the resonant wavelength at each interrogation point varies.

In an earlier paper, we reported photoacoustic mesoscopic imaging *ex vivo* using miniaturized photothermally-tunable fiber optic FPI sensors (Chen et al., 2020). During image acquisition, the sensor’s cavity length is dynamically tuned by a beam of heating light, enabling the resonant wavelength to be quickly adjusted to lock the sensor at its maximal sensitivity (tuning range up to 5 nm). Such a tuning mechanism enables sensor interrogation at a single wavelength, leading to reduced overall cost and potential multiplexing capability. In this work, we further advanced the sensor for higher sensitivity and better acoustic response, and then packaged and assembled the sensor into a scanning PACT system. Resolution test was performed and mice undergone craniotomy were used for brain imaging. We applied the tunable FPI sensors to image *in vivo* mouse brain structure and function with high resolution. To demonstrate the fidelity of the imaging result, main arteries and veins were labeled referring to existing cerebrovascular atlas.

## Materials and Methods

### Imaging System Setup

The system setup was typical of a single element PACT imaging device. A pulsed Ti:Sapphire laser (LOTIS TII LS-2145-LT150) was used for excitation at a repetition rate of 10 Hz, providing wavelengths from 700 to 900 and 1,064 nm. The laser beam was expanded by an expander (LINOS) and reflected onto the mouse brain. The beam profile was shaped by reflecting the beam twice with mirrors. This approach was applied to (1) attenuate the pulse energy as the expanded beam diameter was larger than the mirrors’ diameter thus part of the beam was chopped; (2) As the rim of the Gaussian beam profile was chopped, the illumination became more uniform. The illumination spot size was approximately 2 cm × 2 cm and the intensity at the skin surface was about 20 mJ/cm^2^ at 1,064 nm and less than 4 mJ/cm^2^ at 800 nm. Our FPI sensor was mounted on a xy-stage for raster scan. The stages consisted of two orthogonal motors, for the motion along the *y*-axis we used a voice coil stage (VCM TECH, OWS120-25) with 1μm resolution. A linear motor (Yokogawa LM110-1N-010AN) moved along the *x*-axis with 2 μm resolution. Thanks to the low repetition rate of the laser, the motor was able to stop before each laser shot and move to the next point immediately after the current data acquisition. Each movement had a step size of 100 μm along either *x*- or *y*-axis, forming a grid of 128 × 128 sample points. The acquisition time was 1,639 s (about 30 min). No average was involved during data acquisition. Currently the imaging speed was limited by the laser repetition rate. A mouse with the top portion of the skull removed was placed in a homemade mouse holder. A shallow water tank (depth was less than 3 mm) with polyethylene (PE) film stretched at the bottom was placed above the mouse head for acoustic coupling. Ultrasound coupling gel was used for acoustic coupling between the mouse head and the membrane under the water tank.

### Ultrasound Sensor Based on FPI

**FIGURE 1:**
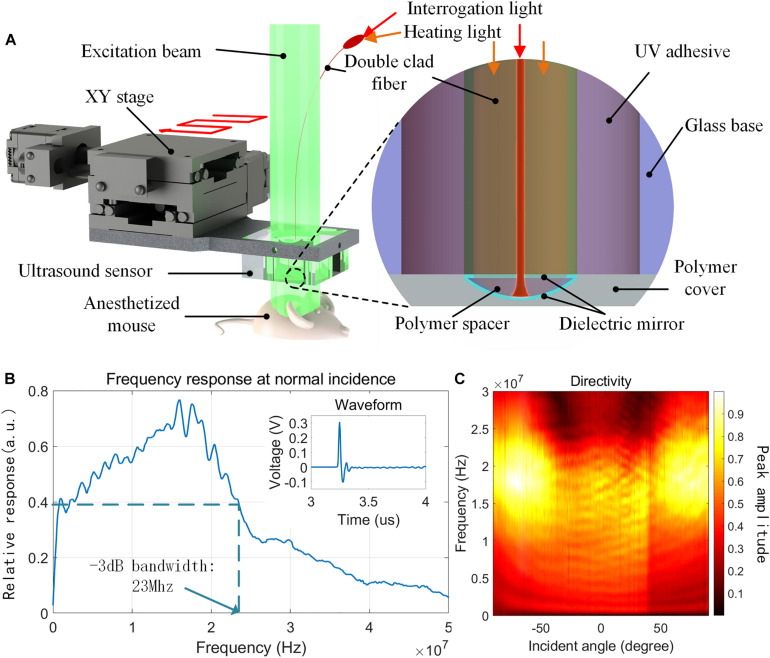
Imaging setup and sensor characterization: **(A)** schematic of the imaging system setup and the sensor structure. The mouse and the excitation beam were static during imaging while the sensor was raster-scanned by the XY stage. The fiber sensor was inserted into the hole of the glass base and fixed by UV-curing adhesive. **(B)** Frequency response at normal incidence obtained by Fast Fourier Transform (FFT) of the corresbonding temporal response shown in inset. The –3 dB bandwidth is measured using the dashed lines. **(C)** Frequency response of the FPI sensor under varying incident angle from –90 to 90°.

Our optically tunable FPI sensor has been reported (Chen et al., 2020). Briefly, the sensor was a high finesse Fabry-Pérot cavity fabricated on the cleaved endface of a double clad fiber. The FPI consisted of two dielectric mirrors and a polymer spacer in between, as shown in Figure 1A. The mirror close to the fiber end was transparent at 650 nm while highly reflective at 1,550 nm. The polymer used for fabricating the sensor cavity was doped with a metal complex dye which was highly absorptive at 650 nm, enabling strong photothermal effect. The coefficient of thermal expansion (CTE) of the cured polymer was on the order of 10^–4^, effectively converting a change of temperature into a change of the optical path length (OPL), thus enabling active tuning of the sensor. The tuning speed could reach 10 nm/s (see Supplementary Figure 1 for details). The core of the fiber was used to deliver the 1,550 nm interrogation light, and the inner cladding was used to deliver the 650 nm heating light. The two laser beams were merged through a fiber coupler (Thorlabs, DC1300LEFA). The fiber-based optical path exhibited high stability and flexibility, making it easier for interrogation and integration. The power of the heating laser was controlled by a proportional integral derivative (PID) control algorithm to stabilize the OPL corresponding to the interrogation wavelength. Before interrogation, the heating power was increased slowly until the DC signal of the photodetector reached the working point, which subsequently triggered the PID control. This process usually took 10 s. The stability of this control mechanism is shown in Supplementary Figure 3. In this application, the fiber sensor was fixed on a glass block and covered by an external polymer layer to protect the sensor during the scanning process.

In this work we tested the response and the directivity of the sensor again since both the sensor and the testing platform have been improved since the earlier publication (Chen et al., 2020). Broadband plane-wave acoustic signal was generated by a thin gold film photoacoustically and calibrated by a Polyvinylidene fluoride (PVDF) hydrophone (Precision Acoustics, NH0200). An 8 mm thick polymethyl methacrylate (PMMA) plate with a diameter of 25 mm was used as the substrate. The coated layer consisted of a layer of 5 nm thick chromium followed by a 100 nm thick gold layer. A diode pumped solid state laser (ZK-Laser SNS-AMP-HE-2) with pulse duration of 2 ns and wavelength of 532 nm was used to excite the gold film through the PMMA plate. The light profile on the gold film was Gaussian shaped with a 1/e^2^ diameter of 9 mm. Theoretically, such acoustic source was broad enough to calibrate our FPI sensor (Rajagopal and Cox, 2020). The results of the temporal response and directivity are shown in Figures 1B,C, respectively. At normal incidence, the temporal waveform still shows a small tail corresponding to the rapid drop of the response beyond 20 MHz. The −3 dB bandwidth of the sensor could be roughly estimated to be 23 MHz. The directivity test was performed using the same acoustic source described above and a direct drive motor (Yaskawa SGMCS-04C3B11). The optical sensor was mounted carefully on the motor that the sensor tip aligned with the rotation center. Totally 181 waveforms of detected signal with incident angle from −90 to 90° were captured with at an interval of 1°. The directivity map exhibits an angular coverage close to 180° over the entire 23 MHz bandwidth. Noise-equivalent pressure (NEP) was also measured using this acoustic source to be 65.8 Pa over the full bandwidth.

### Image Reconstruction and Data Processing

We used a 3D universal back projection algorithm to reconstruct the image (Xu and Wang, 2005). Hilbert transform was performed along the *z*-axis to eliminate negative artifact, making the image more suitable for 3D visualization. CUDA library was used to accelerate the algorithm. The GPU model was NVIDIA TITAN RTX and typically reconstructing an image of 360 × 240 × 360 pixels from 16,384 waveforms would take 15 s (excluding the time for reading the data from the disk). A double speed of sound (SOS) reconstruction method was used for images with large depths. The area of higher SOS was modeled as an ellipsoid whose shape was manually adjusted to match the surface profile of the mouse head. SOS inside and outside the ellipsoid was set manually. The time of flight of the acoustic waves was calculated analytically. The double SOS reconstruction process usually took 30 s with the same parameters of single SOS.

The reconstructed image was rendered and displayed by MATLAB. Further image processing, including fluence compensation and image segmentation, was performed in MATLAB as well.

### Animal Preparation

The animal was prepared by Beijing Vital River Laboratory Animal Technology Co., Ltd. All experimental procedures were in accordance with the National Institutes of Health Guidelines on the Care and Use of Laboratory Animal. The permission code was P2019090. The animal species used in this study was the NU/NU nude mice. Craniotomy was done for each mouse to remove the top portion of the skull. The scalp was sutured back after the surgery and the mouse was bred alone for at least 7 days before the imaging experiment. The mouse can survive for months and was suitable for long-term observation. During imaging, the mouse was anesthetized by inhaling isoflurane of 1.5%. Hair on the scalp was removed before imaging to avoid bubbles. The mouse holder integrated a customized stereotaxic frame to fix the head, which helps reduce the motion blur during the scanning process.

## Results

### System Resolution

**FIGURE 2:**
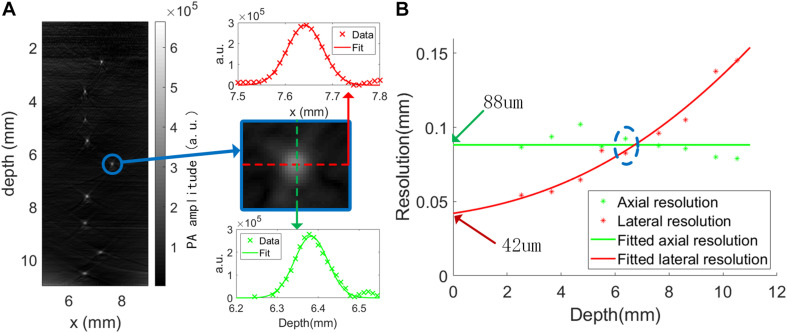
Resolution measurements: **(A)** image of nine 9 μm diameter tengsten wires. The resolution measurement process is illustrated with the bright spot inside the blue circle. The horizontal and vertical cross sections of the spot are shown in “**×**” and were fitted by Gaussian functions. The FWHM was then estimated. Red indicates lateral direction and green indicates axial direction. **(B)** Resolution as a function of imaging depth. The discrete points correspond to the bright spots in **(A)**.

Resolution test was performed by imaging 9 μm diameter tungsten wires, which were considered much smaller than the system resolution. Nine wires were placed parallel to the y axis at different depths. An *x*-*z* slice of the reconstructed image is shown in Figure 2A. The profile can be seen as the line spread function (LSF) of the system. The profile of each tungsten wire was fitted with Gaussian function to calculate its full width at half maxima (FWHM), which is considered as the resolution of the corresponding axis. The results are plotted and fitted in Figure 2B, the axial resolution was uniformly around 90 μm inside the field of view while the lateral resolution increased with imaging depth. This is because the axial resolution is mainly dependent on the bandwidth of the sensor but the lateral resolution is dependent on the bandwidth, the effective detection aperture, as well as the spatial sampling frequency. The reduction of lateral resolution with depth is mainly due to the reduced detection aperture. For our sensor has a large angular coverage (nearly 180°), the axial resolution and the lateral resolution should be the same at depth of zero. It is suspected that the doubled axial resolution was caused by ringing effect of the sensor and worsen by the Hilbert transform along the *z*-axis during reconstruction. Considering that the excitation pulse duration was 25 ns, the elastic relaxation time defined minimum resolution was around 38 μm, very close to the best estimated resolution (around 42 μm), so we suspect that the bandwidth measured in Figure 1B is limited by the optical pulse duration more than the real bandwidth of the sensor.

### *In vivo* Mouse Brain Imaging

**FIGURE 3:**
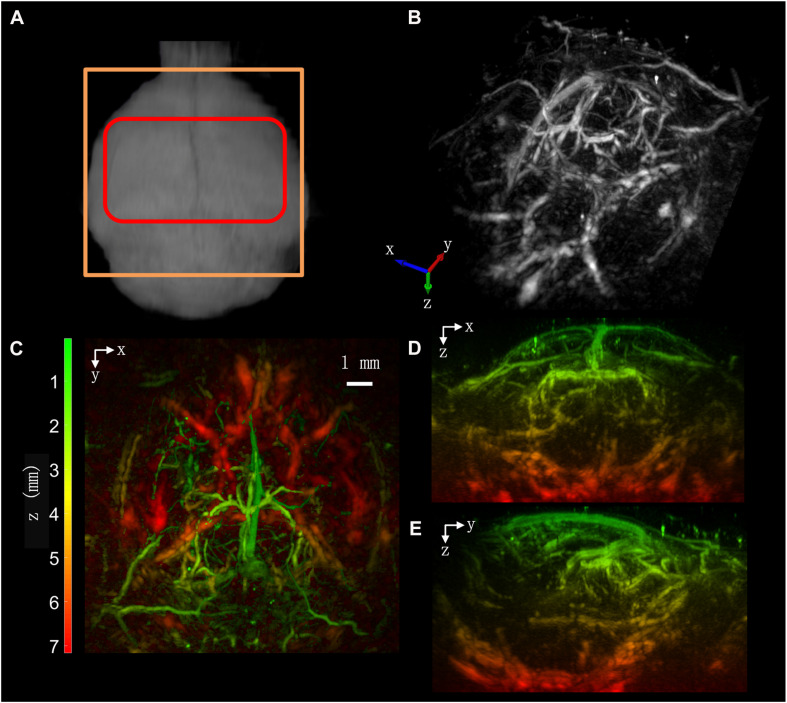
Imaging results: **(A)** imaging area (shown in orange) and position of the cranial window (shown in red). **(B)** MIP of the 3D imaing result from inclined top view. **(C)** MIP of the imaing result in x-y plane. **(D)** MIP in x-z plane. **(E)** MIP in y-z plane. **(C–E)** Are color coded along the z direction.

Figure 3 demonstrates the imaging capability of our system. In Figure 3A, the MRI image of the mouse brain is shown to illustrate the positions of the scanning range and the cranial window. The red box shows the position of the cranial window within which acoustic attenuation and distortion were low. The window was opened from the herringbone and extended about 5 mm upward. The lateral width of the window was up to approximately 9 mm, almost covering the whole parietal bones. The orange box as large as 12 × 12 mm indicates the FOV of the image, which was slightly larger than the cranial window. Note that the depth of FOV could be extended to 10 mm if needed.

**FIGURE 4:**
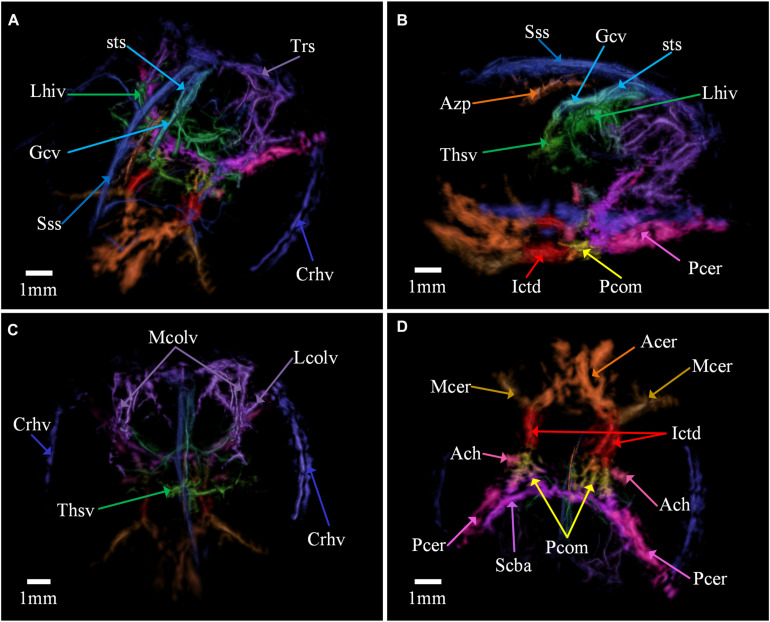
The segmentation result of the cerebovascular structure. **(A)** The same view with Figure 3B. **(B)** Left view. **(C)** Horizontal slice plane at lateral-medial of the brain, inferior view. **(D)** Bottom view, showing the circle of Willis. Sss, superior sagittal sinus; Trs, transverse sinises; Crhv, caudal rhinal vein; Gcv, vein of galen; sts, straight sinus; Lhiv, longitudinal hippocampal vein; Thsv, thalamostriate vein; Mcolv, medial collicular vein; Lcolv, lateral collicular vein; Ictd, internal carotid artery; Acer, anterior cerebral artery; Mcer, middle cerebral artery; Pcer, posterior cerebral artery; Ach, anterior choroidal artery; Azp, azygos pericallosal; Pcom, posterior communication artery; Scba, superior cerebellar artery.

To present image features in 3D better, the image was rendered in grayscale as shown in Figure 3B. The main veins and arteries can be seen clearly in this image. Because of the high sensitivity of the optical ultrasound sensor, the imaging depth of our system could cover the whole brain and the circle of Willis can be seen in this image (see Figure 4D) for details. The veins within 5 mm depth are imaged with high quality. The images are shown with a depth-coded color scheme and maximum intensity projection (MIP). Three different views along the three axes are shown in Figures 3C–E.

Although we tried hard to remove bubbles inside the FOV, it’s difficult to remove them all. There were still some ultra-bright spots above the main structure, bringing down the dynamic range and introducing artifacts into the image. These bubbles were mainly trapped inside the ultrasound coupling gel, the large viscosity of which prevented effective removal of the bubbles.

The scanning surface was very close to the mouse head (less than 1 mm), still, our system suffered from the limited view problem. The small diving vessels on the cortex were almost entirely invisible. The cranial window with limited aperture size complicated the situation since many vessels are very close to the skull. The resolution at large depth was not as good as expected. Besides the reduced effective aperture due to the cranial window, the breathing motion right above the oral cavity also conspired to worsen image resolution at deeper regions.

### Segmentation of Cerebral Vascular

Cerebrovascular segmentation is essential for the study of brain structure and function, and is important for the analysis, diagnosis, and treatment of blood-related brain diseases. The cerebrovascular structure of mouse has been investigated extensively, and the atlas was made by CT or MRI of a fixed *ex-vivo* mouse brain, or by sequentially slicing and imaging using optical microscopy then stitching the images for 3D reconstruction. By comparing our images with the existing established ones, most of the big vessels in the image, especially arteries in deep tissue, were found and labeled, showing the high fidelity of our imaging results. The annotation of arteries and veins comes from an existing atlas made by micro-optical sectioning tomography (Xiong et al., 2017).

Because the cranial window was just above parietal temporal lobe, most arteries shown in the image are supplied by the internal carotid arteries (Ictd), the red arteries at the bottom of the 3D image (see Figures 4B,D). Its major branches include the anterior (Acer), middle cerebral arteries (Mcer) and anterior choroidal artery (Ach) as well as parts of the posterior cerebral arteries (Pcer), labeled in orange, brown, fuchsia and pink, respectively. The two anterior arteries go anteriorly and fuse together and then split into three branches, as shown in Figure 4D. The middle one goes a little upward and eventually connects with the azygos pericallosal artery (see Figure 4B). Unfortunately, this connection relationship cannot be observed because of the limited view problem. The two Mcers are flattened on the brain surface and only the bottom part can emit acoustic waves upward because skull blocks the signals coming from its branches, so only the initial part of the Mcers can be observed. The posterior communicating artery (Pcom) is a very short artery connecting Pcer and the superior cerebellar artery (Scba), colored in yellow. Acer, Mcer, Pcer, Ictd, and part of Scba together form the circle of Willis, as shown in Figure 4D.

The vein system upward is clearer and the large veins and sinuses like the superior sagittal sinus (Sss), straight sinus (sts) and vein of Galen (Gcv) can be recognized in the image, see Figures 4A,B. The signals from the transverse sinuses (Trs) were blocked by the skull but the structure is still visible. The caudal rhinal vein (Crhv) lies in the lateral-medial of the brain and connects with Trs. The medial collicular veins (Mcolv) and lateral collicular veins (Lcolv) join tighter as well, as shown in Figure 4C. Joined with Lcolv, the longitudinal hippocampal vein (Lihv) locates inferior to Gcv. And thalamostriate vein (Thsv) is also observed anterior to Lihv and joins Gcv.

### Dual Wavelength Imaging of Mouse Brain

To show the multispectral imaging capability of the system, PA images excited at 1,064 and 800 nm were acquired for the same mouse. The scanning system guaranteed that the positioning error of the two images was less than 2 μm, much smaller than the resolution of the system. The data acquisition of 800 nm illumination started immediately after that of 1,064 nm illumination. The total imaging process took about an hour.

**FIGURE 5:**
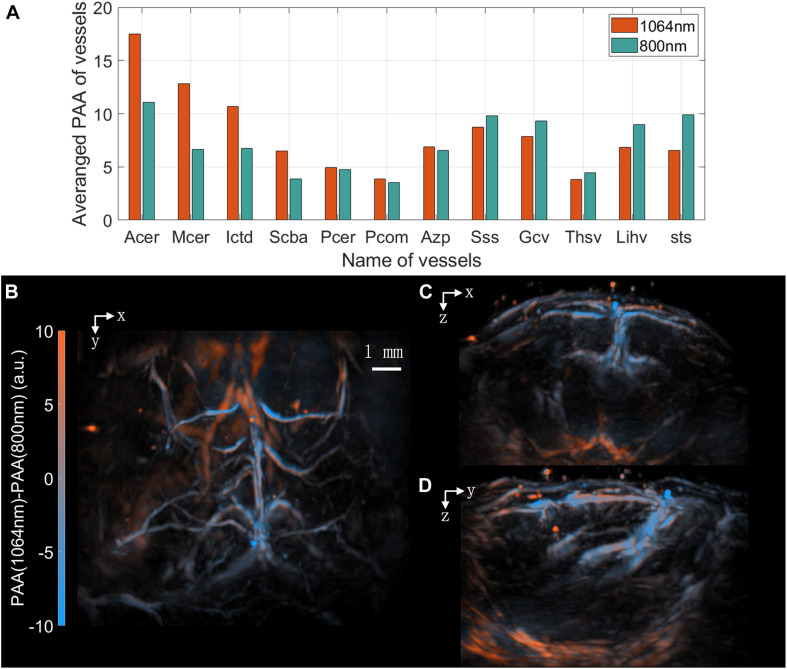
Double wavelength imaging results. **(A)** Averaged PAA of different vessels. Arteries has larger PAA at 1,064 nm and veins has larger PAA at 800 nm. **(B)** MIP of the sum of the PA images excited at 1,064 and 800 nm in the x-y plane. **(C)** MIP in the x-z plane. **(D)** MIP in the y-z plane. **(B–D)** Are color coded by the difference of the two PA images excited at 1,064 and 800 nm.

Then, PA images were reconstructed and then the outline of the mouse head and the vessels were segmented. The profile of the head was used in a Monte-Carlo simulation to calculate the optical fluence at the two wavelengths (Yu et al., 2018). Based on these results, fluence compensation was applied. And the segmentation of vessels was used to calculated the average photoacoustic amplitude (PAA) of each vessel, as shown in Figure 5A. All the vessels listed can be found in 3.3. At the wavelength of 800 nm, oxyhemoglobin and deoxyhemoglobin have the same absorption coefficient thus they contribute to the PAA equally. On the other side, absorption coefficient of oxyhemoglobin is larger than that of deoxyhemoglobin at 1,064 nm, so vessels with more oxyhemoglobin will have larger PAA at 1,064 nm. Making it easier for comparison, both images at 1,064 and 800 nm were normalized by their total energy, so arteries with more oxyhemoglobin tended to be brighter at 1,064 than 800 nm while veins with less oxyhemoglobin tended to be brighter at 800 than 1,064 nm. Color-coded by the difference of the two images of 1,064 and 800 nm, the imaging results are shown in Figures 5B–D in three different views. Most of the arteries and veins can be distinguished clearly as arteries are reddish and veins are bluish in these images. Interestingly, the bubbles trapped in the ultrasound coupling gel above the mouse head are red, indicating that the gel has larger absorption coefficient at 1,064 than at 800 nm. This might be attributable to water, the main constituent of the gel, whose absorption is greater at 1,064 nm.

Using the above method, we recovered some information about the concentrations of oxyhemoglobin and deoxyhemoglobin inside the mouse brain, but it was still difficult to calculate the accurate sO_2_. The main reason was the scanning time was too long. One consequence was the body temperature of the mouse changed during the process, causing the image at 800 nm slightly shifted compared to the 1,064 nm one. Though the speed of sound was adjusted accordingly during reconstruction, it was difficult to align the vascular structure pixel by pixel. This problem would result in large error in calculating sO_2_. Another problem was that sO_2_ might change during the scanning process, making the results unreliable.

## Discussion

In this study, we present a PA imaging system as a potential platform for preclinical research of the mouse brain. The system employs a photothermally tunable FPI ultrasound sensor. In-vivo imaging of the mouse brain was demonstrated. The FPI sensor equipped with a temperature-controlled spacer was able to tune the resonant wavelength using a 650 nm diode laser. Such a sensor did not compromise neither optical nor acoustic properties. The photoacoustic imaging system using this sensor was then applied in cerebrovascular structure imaging of the mouse brain. The broadband, highly-sensitive and wide-angular-coverage signal detection provided high resolution and large imaging depth. The high-fidelity image was segmented, rendered and compared with existing literature. The capability of structural imaging further paves the way for the study of brain functions, for example, high fidelity cerebrovascular map can be used as the structural prior in inverse problems when spatial resolution is sacrificed for temporal resolution. In addition, we demonstrated the system’s potential for functional imaging by an experiment involving dual-color excitation.

It is a common practice to remove or thin the skull for ultrasound or photoacoustic imaging, as the skull will cause strong attenuation and distortion of the acoustic signal. In this study, although the mouse model with the long-term cranial window allows convenient longitudinal study, the removal of the skull poses potential risks of inflammation and abnormal intracranial pressure. How such potential side effects could perturb biological study needs further investigation. Despite the invasiveness, imaging with higher signal-to-noise ratio and better fidelity is sometimes preferred. Nevertheless, our system is capable of imaging mouse noninvasively, at the cost of reduced image quality.

The PA mesoscope provides a relatively economical solution to high quality 3D imaging of the mouse brain. Electrical transducers are suboptimal for PA imaging due to their narrow bandwidth and limited angular coverage. Moreover, the transducers are typically bulky and opaque, making mechanical scanning and optical illumination challenging. To achieve high-quality 3D imaging, specially designed transducers, such as grouping of transducers for bandwidth extension, are employed. Existing optical sensors with the needed sensitivity, bandwidth and angular coverage is also costly. Our solution can replace the tunable interrogation laser (usually exceeding $30,000) by a fixed wavelength laser and a heating laser diode (costing less than $6,000 in total). Furthermore, the sensor can be batch-fabricated. Because the FPI sensor is simple, robust and miniaturized, it’s potentially suitable for multiplexing.

Currently, the scanning time of nearly 30 min is too long for certain applications, and multiwavelength imaging takes even longer. Although respiratory gating technologies can be used to mitigate the effect of breathing motions as in micro-CT and MRI, the long scanning time still prevents most functional imaging applications. Now this problem is mainly due to the low repetition rate (10 Hz) of the laser, and using a faster excitation laser (1 kHz for example) will help decrease the scanning time to within 20 s. Moreover, sensor multiplexing will further reduce the data acquisition time to enable fast 3D functional brain imaging using photoacoustics. Nevertheless, interrogation of multiplexed optical sensors using a single light source requires accurate control of the sensors’ OPL during fabrication. Thermally-tunable sensors with extended tuning range (up to 20 nm) are under development to relax the requirement for fabrication accuracy.

## Data Availability Statement

The raw data supporting the conclusions of this article will be made available by the authors, without undue reservation.

## Ethics Statement

The animal study was reviewed and approved by the Institutional Animal Care and Use Committee of Beijing Vital River Laboratory Animal Technology Co., Ltd.

## Author Contributions

CM and WH conceived and designed the project and coordinated activities from all authors. BC designed and fabricated the FPI sensor. BC and YC tested and characterized the sensor. YC designed and built the imaging system and performed process to the data. YC, LY, and TY used the system to acquire data of mouse brain. YC and LY labeled the vascular system. YC, BC, CM, and MS wrote the manuscript.

## Conflict of Interest

The authors declare that the research was conducted in the absence of any commercial or financial relationships that could be construed as a potential conflict of interest.
