# Supplementary material for: Photoacoustic Mouse Brain Imaging Using an Optical Fabry-Pérot Interferometric Ultrasound Sensor
Source: Front Neurosci. 2021 May 17;15:672788. doi: 10.3389/fnins.2021.672788 (PMC8165253; doi:10.3389/fnins.2021.672788)
Supplement: Supplementary file 1 [file Data_Sheet_1.PDF]

Supplementary Material

1 Supplementary Figures

Supplementary Figure 1. Scheme of the fiber coupler.

Supplementary Figure 2. Tuning speed of the thermal tuning method.

Supplementary Figure 3. Unsmoothed data of tuning speed.

Supplementary Figure 4. Stability at resonance wavelength.

1.1 Supplementary Figures

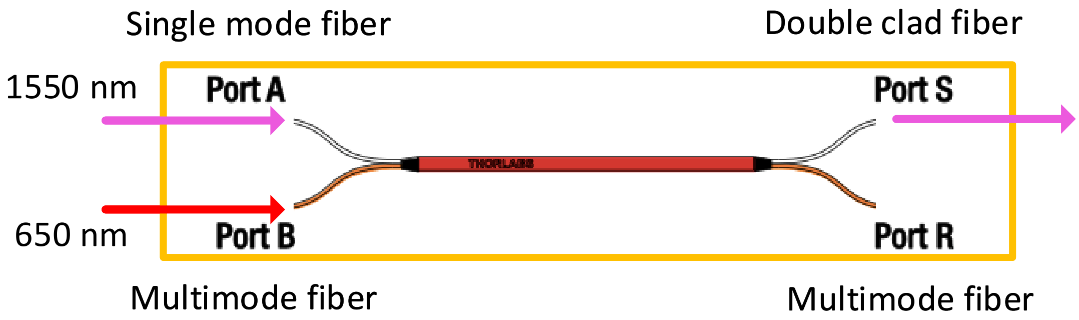

Supplementary Figure 1. The fiber coupler (Thorlabs, DC1300LEFA) has a double clad fiber output port (PORT S)—the 650 nm light from one input port (PORT B) is coupled to its inner cladding while the 1550 nm light from another input port (PORT A) is coupled to its core. As a result, the 650 nm heating light and the 1550 nm interrogation light can be delivered simultaneously in the same fiber to illuminate the FPI. The 650 nm light coupling efficiency was measured to be 29%. The rest of the 650 nm light was delivered to PORT R which can be connected to a power

meter. As the ratio of 650 nm light power from PORT S to PORT R was a constant (measured to be 41%), we monitored the heating power by measuring the output of PORT R in some experiments.

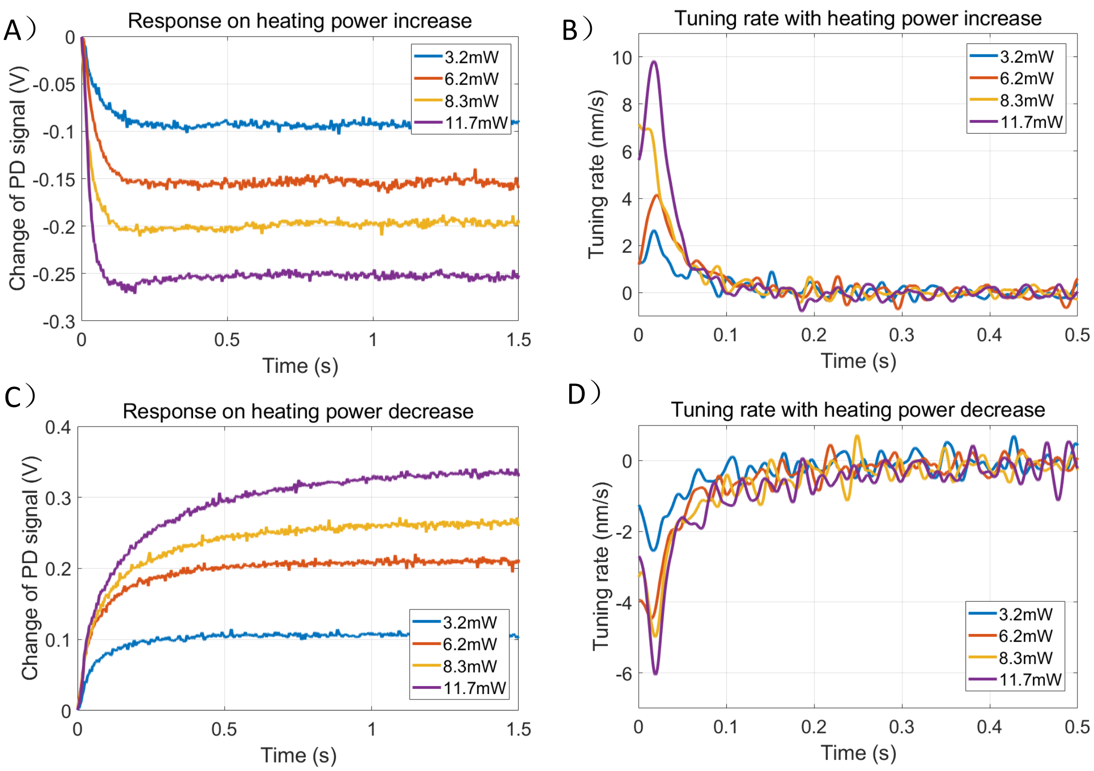

Supplementary Figure 2. Tuning ability of the sensor. Heating power changes was applied at t=0. (A). Changes of the PD signal after increasing the heating light power. The legend represents the increment of heating power at t=0. (B). Smoothed tuning rate corresponds to (A). (C). Changes of the PD signal after decreasing the heating light power. The legend represents the decrement of heating power at t=0. (D). Smoothed tuning rate corresponds to (C). the PID program was shut down after stabilized at resonance wavelength, the sensor was kept steady in a constant-temperature bath to minimize extra shift. Then the heating power was increased or decreased rapidly to tune the sensor. The output signal of the PD was recorded as the step response, as shown in Fig. R 1 (A) and (C). Heating light power was monitored by measuring the output of PORT R of the coupler and scaled by factor 41% (See captions for Supplementary Figure 1). The voltage signal was converted to wavelength and then the tuning speed. The data was quite noisy (see Supplementary Figure 3). Despite the electrical noise induced by power supply, we think the low frequency noise also comes from the circulator. To make it clear, a 25 ms width gaussian window was applied to smooth the speed curve and the results are shown in Fig. R 1 (B) and (D). It can be observed that the tuning

speed was on the order of 1 nm/s, and the temperature decreasing process was slower indeed, but still on the same order, especially when the change was small.

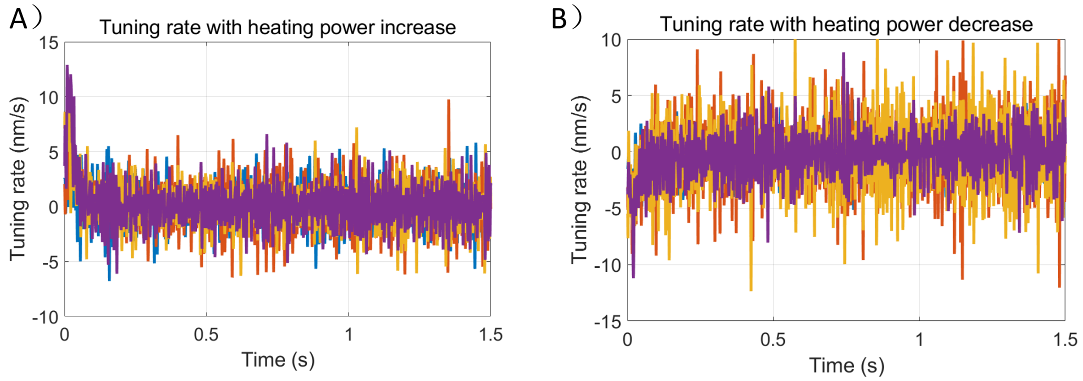

Supplementary Figure 3. Unsmoothed data of tuning speed. (A). Tuning rate corresponds to Supplementary Figure 2. (A). (B). Tuning rate corresponds to Supplementary Figure 2. (C).

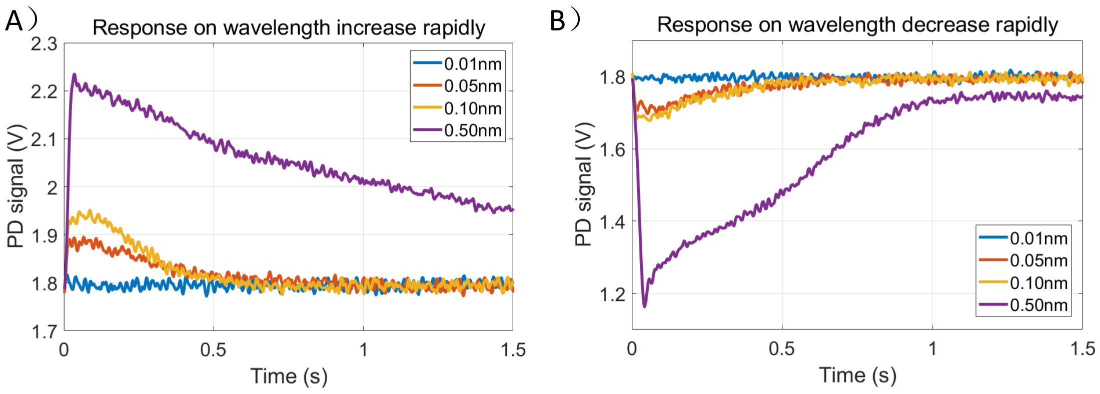

Supplementary Figure 4. Stability of the sensor. (A). Sensor response when increasing interrogation wavelength rapidly at t=0. The legend shows the increment of the wavelength. (B). Sensor response when decreasing interrogation wavelength rapidly at t=0. The legend shows the decrement of the wavelength. Fast tunable laser was used for interrogation. After stabilized at resonance wavelength by thermal method, interrogation wavelength was changed rapidly to create a step disturbance. Meanwhile, the response of the PD signal was recorded. For perturbation like the 0.01 nm changes, the system handled well. However, when facing larger disturbance, it took some time to go back to the working point (less than 0.5 s). For the 0.5 nm test, we think the interrogation wavelength has went out of the linear region so it took even longer. Actually, such huge disturbance was rare in the real imaging process, as we slowly added up the heating power until the DC signal of PD approaches the desired voltage before the PID algorithm started up. We are not experts in automation, the stabilizing time and the noise in response curve indicate there are still some works to do on the stability. While the environment of the sensor was not that hard during imaging, the current stability was competent for our system.
